# When irrelevant information helps: Extending the Eriksen-flanker task into a multisensory world

**DOI:** 10.3758/s13414-020-02066-3

**Published:** 2020-06-08

**Authors:** Simon Merz, Christian Frings, Charles Spence

**Affiliations:** 1 Department of Psychology, Cognitive Psychology, University of Trier, Universitätsring 15, 54286 Trier, Germany; 2 Department of Experimental Psychology, University of Oxford, Oxford, UK

**Keywords:** Flanker task, Information processing, Vision, Touch, Audition, Multisensory processing, Crossmodal attention

## Abstract

Charles W. Eriksen dedicated much of his research career to the field of cognitive psychology, investigating human information processing in those situations that required selection between competing stimuli. Together with his wife Barbara, he introduced the flanker task, which became one of the standard experimental tasks used by researchers to investigate the mechanisms underpinning selection. Although Eriksen himself was primarily interested in investigating visual selection, the flanker task was eventually adapted by other researchers to investigate human information processing and selection in a variety of nonvisual and multisensory situations. Here, we discuss the core aspects of the flanker task and interpret the evidence of the flanker task when used in crossmodal and multisensory settings. “Selection” has been a core topic of psychology for nearly 120 years. Nowadays, though, it is clear that we need to look at selection from a multisensory perspective—the flanker task, at least in its crossmodal and multisensory variants, is an important tool with which to investigate selection, attention, and multisensory information processing.

It is almost half a century since B. A. Eriksen and C. W. Eriksen first introduced a simple yet elegant task with which to investigate information processing and selective attention in human cognition (see B. A. Eriksen & C. W. Eriksen, 1974). These researchers were interested in probing the limitations of the human ability to selectively attend to specific sensory events. Their curiosity was sparked by a surprising discovery—namely, that distracting information still influenced task performance when participants were given ample time in which to focus their attention on the location where a target stimulus would subsequently be presented (Colegate, Hoffman, & Eriksen, 1973; Eriksen & Hoffman, 1972, 1973). This finding was taken to indicate that the ability to focus attention on a predefined spatial location was limited, and this seemed worthy of further exploration. Yet, as their previous task consisted of a relatively crowded stimulus display with letters arranged in a circular display and the presentation of multiple distractors, the task was simplified. The task-relevant stimulus was defined by its spatial location, and three distracting stimuli were presented on either side of the target, flanking the central target stimulus (e.g., BBBABBB), which subsequently led to this being named the *flanker task*. Different conditions were investigated in which the target–distractor relation was systematically varied and the participant’s response to the various conditions compared. The rationale was simple: If the response to the target stimulus changed as a result of the identity of the distractor stimulus, then this meant that the distractor must still have been processed to a certain extent. This therefore indicates that the attentional focus could not have been precise enough to exclusively focus on one specific location.

The influence of this classic work cannot be overstated, as shown most prominently by the 6,400 citations this paper has received as of May 2020 (according to Google Scholar). Interestingly, selective attention was mostly investigated and discussed in the visual modality, and several theoretical explanations such as the spotlight (e.g., Norman, 1968; Posner, 1980; Posner, Synder, & Davidson, 1980) and zoom lens (e.g., Eriksen & St. James, 1986; LaBerge, 1983) metaphor/model are implicitly visual, at least in terms of the language used. Yet, in the 1990s, interest started to grow in the study of the mechanism(s) underlying selective attention in settings other than those that were exclusively visual. It has long been known that we are able to attend selectively to specific information while ignoring other irrelevant information in the auditory modality (e.g., just think about the classical cocktail party phenomenon when you are trying to listen to what your friend is saying while at a noisy party, e.g., Cherry, 1953; Shinn-Cunningham, 2008) or touch (e.g., just imagine holding a tennis racket in your hand while ignoring other sensory information, like the feel of the blister on your foot). Even more, our everyday life is fundamentally multisensory in nature. Selective attention is not just needed in unisensory settings (assuming, that is, that such situations even exist), but also in crossmodal (e.g., reading a book while ignoring the buzzing of the phone) and multisensory (e.g., watching and listening to the news on TV while trying to ignore background noise) situations.

This review focuses specifically on those investigations into the mechanisms of selection that have investigated crossmodal (the interplay between at least two modalities during selection) and multisensory (selection between stimuli which are themselves composed of features from at least two sensory modalities) selection. That is, selection situations in which at least two sensory modalities are. In a first step, the experimental logic of the flanker task is outlined, detailing its advantages and highlighting its flexibility and adaptability as far as the investigation of human information processing is concerned. In a second step, the central studies that have used the flanker task are discussed, highlighting in particular those studies that have been conducted across different senses. Thereafter, the focus will be broadened out in order to highlight the more general interplay between attention and selection in a multisensory world. Furthermore, we also detail how the flanker task and successive adaptations of the basic underlying paradigm may, in future research, be used to tackle outstanding theoretical questions in our multisensory world.

## The flanker task

In the classic version of the flanker task, only a small set of possible target stimuli are chosen. These need to be discriminated by the participant and responded to with one of two possible, typically manual, responses. More or less simultaneous with the target stimulus, one (or more) distractor stimuli are presented. The distractors are possibly also chosen from the same set of target stimuli, and therefore mapped on to a specific response. Alternatively, the distractor stimuli are chosen from a new stimulus set bearing no relation to the possible responses or, on occasion, no distractor is presented at all. This experimental design constitutes the core of the flanker task, as it allows for the analysis of the existence of any influence of the distracting information. In addition, the comparison of different trial types allows for an analysis of the depth to which the distracting information is processed, and thus helps to answer the question about early or late selection (e.g., see Lavie & Tsal, 1994; Pashler. 1994a, 1994b, 1998).

### The core of the flanker task: The relation between target and distractor

**Fig. 1:**
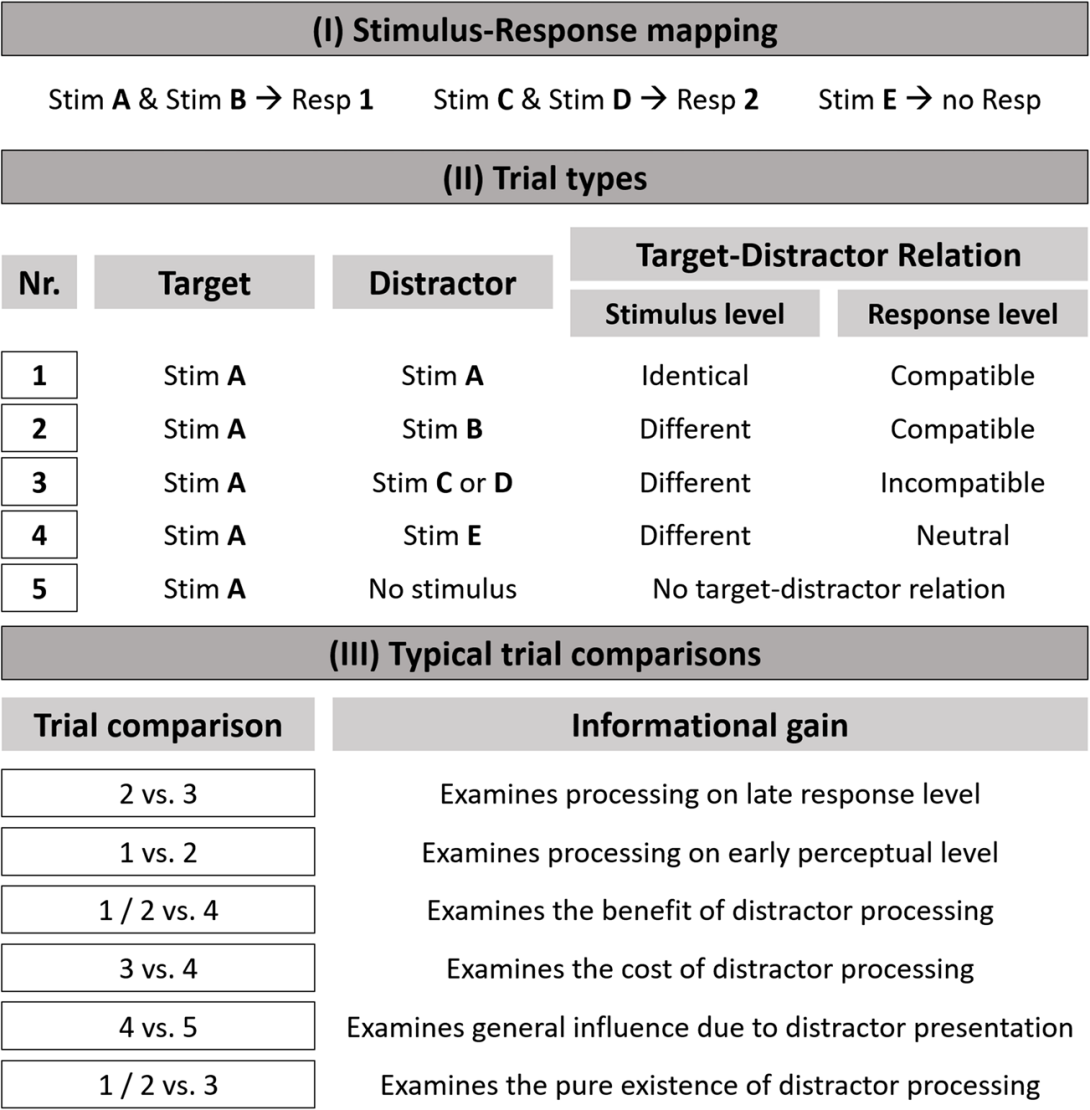
Overview of the underlying logic of a typical flanker task. **I** The 4 × 2 SR mapping used in many studies and the basis for the trial types and trial comparison presented here. **II** The five most typical trial types used within a 4 × 2 SR mapping flanker task. **III** Typical trial comparisons and what they tell researchers about the processing of irrelevant information. For more information, see the main text. Stim = stimulus; Resp = response

The central manipulation in the flanker task is the systematic pairing of different distractor and target stimuli. In its classical form, a 4 × 2 stimulus–response (SR) mapping is used. This is where four different target stimuli (Stim **A**-**D**) are mapped on to two different responses (Stim **A** & Stim **B** ➔ Resp **1**; Stim **C** & Stim **D** ➔ Resp **2**; see Part **I** of Fig. 1). At least three different trial types can be presented using this 4 × 2 SR mapping (see Part **II** of Fig. 1 for a schematic illustration of the most prominent trial types in classical flanker task designs). The target–distractor relation can be described at two different levels: one related to the stimulus and the other related to the response. At the former level, the target and distractor can either be identical (Trial Type Nr. **1**; target & distractor = Stim **A**; although note that in this case, no selection is really needed; cf. Chan, Merrifield, & Spence, 2005) or different (all other trial types; target = Stim **A**, distractor ≠ Stim **A**).

Additionally, the target–distractor relation can be described on the response level. If both the target and distractor are identical (Trial Type Nr. **1**), this necessarily also means that both of the stimuli would be linked with the same response, and thus they are response compatible. Yet there is another possibility that the target–distractor relation is response compatible while the stimuli themselves are not identical. That is, the target and distractor are different, but are nevertheless mapped on to the same response (Trial Type Nr. **2**; target = Stim **A**, distractor = Stim **B**). The third possibility is that the target and distractor are response incompatible that is, they are mapped to different responses (Trial Type Nr. **3**; target = Stim **A**, distractor = Stim **C** or **D**). Additionally, the distractor might not have been mapped on to any response and therefore this trial is a response neutral trial (Trial Type Nr. **4**; target = Stim **A**, distractor = Stim **E**). Alternatively, no distractor is presented at all, and, hence, no selection between stimuli is required (Trial Type Nr. **5**).

The intriguing aspect of the flanker task is that by comparing performance in the different trial types, the processing of distractors can be analyzed (for an overview, see Part **III** of Fig. 1). The most common comparison involves comparing response compatible (Trial Type Nr. **2**) and incompatible (Trial Type Nr. **3**) trials as the difference reflects distractor processing only at the level of response selection. For example, better performance in compatible than in incompatible trials would indicate that the distractor was processed up to the level of response selection. As the target and distractor were perceptually incongruent in both type of trials, differences in performance could only be due to the difference at the response level. Comparing identical (Trial Type Nr. **1**) and response compatible (Trial Type Nr. **2**) trials reveals distractor interference at the perceptual level without any influence of response compatibility. Moreover, response compatible and incompatible trials (Trial Type Nr. **1**–**3**) are compared with neutral response trials (Trial Type Nr. **4**), as this allows for the calculation of the benefits and cost of processing the distractor (although this calculation also has its challenges; cf. Jonides & Mack, 1984). Many further comparisons can be made even using just the original version of the flanker task. Yet, in many published studies, compatible and identical trials are not separated, thereby discarding the virtue of the flanker task to disentangle interference at the level of perception from interference at the level of response compatibility (e.g., see Bossert, Kaurin, Preckel, & Frings, 2014; Geißler, Hofmann, & Frings, 2020, on this issue).

The possibilities of conducting modified versions of this task are manifold. For example, one might be interested in the similarity of the distractor to the target, so one might use different response-neutral conditions the distractors are either more dis/similar to the target (e.g., B. A. Eriksen & C. W. Eriksen, 1974). Or, you might use a more complex stimulus response mapping with different response categories (e.g., manual as well as foot-pedal response) in order to see whether a distractor that indicates a response in the same or different response category is processed differently (cf. Gallace, Soto-Faraco, Dalton, Kreukniet, & Spence, 2008). These examples highlight one of the crucial advantages of the flanker task—namely, its flexibility. It can be adopted to fit the exact needs of one’s specific research question while maintaining the crucial feature that enables one to disentangle different levels of interference processing.

The flexibility of the flanker task opens up a lot of different possibilities for the investigation of information processing, as outlined in the previous paragraph. Yet it is important to bear in mind that changes in the specific experimental environment (e.g., adding or removing specific trials) might, on its own, change the nature of the information processing that is observed. In fact, some studies have shown that targets might be responded to somewhat differently depending on the set of other stimulus configurations presented during the same block of experimental trials (C. W. Eriksen & B. A. Eriksen, 1979; Frings, Merz, & Hommel, 2019; for related evidence in other experimental tasks, see Gau & Noppenay, 2016; Geng, DiQuattro, & Helm, 2017; Mast & Frings, 2014). That is, the relative frequency with which specific trial types are presented as well as the inclusion or exclusion of specific trial types within the same experimental block seems to influence the actual task performance during otherwise identical trials. This might be due to an attentional/informational shift between the competing stimuli or an updating of prior expectations (e.g., Gau & Noppenay, 2016), possibly relating to more general cognitive mechanisms like curiosity exploration (Berlyne, 1960) or mental and behavioral flexibility (Hommel, 2015).

To summarize, the flanker task is well suited to the investigation of the processing of irrelevant information during selection situations. The design is simple and easy to use, with an enormous adaptability to match a wide variety of different research questions. Thus, the flanker task provides a helpful tool with which to gather evidence concerning information processing in many different theoretical contexts (e.g., in the study of memory, Eriksen, Eriksen, & Hoffman, 1986; or the relevance of perceptual load for information processing, Lavie, 1995; Miller, 1991; see also Lavie, 2010). In fact, it tackles an important theoretical question concerning our cognitive system and has therefore sparked much interest throughout different disciplines. The flanker task focusses on the limitations of our cognitive system to actively select what we process. The task addresses the question of the extent to which we process irrelevant information even though we do not need or want to, similarly to the Simon task (Simon, 1990; Simon & Rudell, 1967; for an early review, see Lu & Proctor, 1995) or the Stroop task (Stroop, 1935). The comparison of trials with different target–distractor relations stands at the heart of the flanker task. The flanker task, then, helps researchers to understand the level of processing that any distracting information undergoes. In fact, this possibility was often used in the investigations of distractor processing outside the visual modality, as our review in the subsequent section will show.

## The flanker task across the senses

Following its introduction by B. A. Eriksen and C. W. Eriksen (1974), the flanker task soon became one of the visual standard tasks with which to investigate the ability to selectively attend specific stimuli. Indeed, the early research nearly exclusively used a visual version of the flanker task, including stimulus sets like letters (as in B. A. Eriksen & C. W. Eriksen’s, 1974, original study), arrows (e.g., Bugg, 2008; Nieuwenhuis et al., 2006), or numbers (e.g., Lehle & Hübner, 2008; Notebaert & Verguts, 2006). As far as we are aware, the tactile modality was the first modality in which the flanker paradigm was transferred outside vision (Evans and Craig, 1991, 1992) using motion stimuli. It took a further decade before the first auditory adaptation of the flanker task was published (Chan et al., 2005). To date, the flanker task has proven to be very helpful for the investigation of selection not only within different sensory modalities (for extensive reviews, see C. W. Eriksen, 1995; Spence, in press; Wesslein, Spence, & Frings, 2014c) but also between them.

### Crossmodal distractor processing in the flanker task

In our daily lives, all our sensory systems constantly receive input, and selection has to occur within as well as between the senses. For example, when listening to a great piece of music, or when trying to identify what a friend wrote on our back when we were young (see Arnold, Spence, & Auvray, 2017, for a review), we do not want to be distracted by other information (which therefore often leads us to close our eyes). In fact, the mere sight of a stimulus (distractor and/or target) has an impact on performance in nonvisual flanker tasks, and variants of the flanker task have been developed in which the targets and distractors were presented in different sensory modalities (the crossmodal congruency task; see Spence, Pavani, Maravita, & Holmes, 2008, for a review).

#### The influence of vision on tactile information processing in the flanker task

The first crossmodal study with the flanker task was, to the best of our knowledge, the investigation of the importance of vision (i.e., sight of the stimulus/body) for tactile distractor processing by Driver and Grossenbacher (1996). This research is in the tradition of studies of the influence of vision on the processing of tactile targets (e.g., Heller, 1982; Honoré, Bourdeaud’hui, & Sparrow, 1989; Tipper et al., 1998). For example, visibility/magnification of the forearm has been shown to increase its tactile spatial resolution (measured by a two-point threshold; Kennett, Taylor-Clarke, & Haggard, 2001).

In several experiments, Driver and Grossenbacher (1996) used a tactile version of the flanker task to investigate the influence of vision on tactile distractor processing. The authors used a 2 × 2 SR mapping—that is, two different stimuli (one long vibration vs. two short vibration bursts) were mapped onto two different responses (foot-pedal responses: lifting the heel vs. lifting the toe). The authors presented one vibrotactile stimuli to each hand; one hand was presented with the target stimulus, and the other with the distractor stimulus. Importantly, Driver and Grossenbacher systematically manipulated the spatial separation between the target and distractor hand, the direction of participant’s gaze, as well as participants’ vision of the set-up via blindfold. Independent of any of the experimental manipulations that were introduced, distractor interference was observed. That is, performance in the congruent trials (target stimulus was identical to the distractor stimulus) was better than in the incongruent trials (target stimulus was different from the distractor stimulus), and this was independent of whether the participants had been blindfolded or not. Comparable to evidence from visual flanker experiments (e.g., Fox, 1998; Miller, 1991), distractor interference decreased as the spatial separation increased. Yet this was not the case when gaze was directed towards the distractor, thus indicating a crossmodal modulation by overt spatial attention of vision on tactile distractor processing.

Although Driver and Grossenbacher (1996) observed no difference between blindfolded and nonblindfolded participants, the conclusion that the visibility of the stimuli has no influence on tactile distractor processing would be unjustified. In fact, the usage of a 2 × 2 SR mapping prevented the authors from investigating any influence on the level of distractor processing. In a later study, Wesslein and colleagues (Wesslein, Spence, & Frings, 2014a) once again manipulated the visibility of the stimuli (by occluding either one or both hands), yet they used a 4 × 2 SR mapping in order to differentiate between the perceptual and response level of distractor processing. Comparable to Driver and Grossenbacher, distractor interference was observed in all conditions. Yet, if vision of both hands was prevented, distractor processing only occurred at the perceptual level, whereas if vision (even just of one hand) was enabled, distractor processing occurred on the perceptual as well as response level.

Subsequently, Wesslein, Spence, and Frings (2015) demonstrated that it is not enough simply to see a hand, but that the hand has to be associated with the person (e.g., via the rubber hand illusion; Botvinick & Cohen, 1998; see also Gallace & Spence, 2014) for distractor processing to occur up to the response level. In a related vein, Wesslein, Spence, and Frings (2014b) observed that an impermeable barrier between the target and distractor hand prevented processing up to the response level, whereas a permeable barrier (specifically an empty picture frame) elicited distractor processing up to the response level (for an extensive review of the crossmodal interplay in visuotactile information processing, see Wesslein et al., 2014c). Such results therefore highlight a robust influence of vision on tactile distractor processing. They also highlight the importance of investigating the different processing levels, as crucial factors like visibility of the stimulated location and higher order cognition critically alter the way in which stimuli are processed.

#### The crossmodal congruency task: The crossmodal version of the flanker task

The importance of investigating selection in crossmodal settings can be illustrated with a specific example. Namely, the change in the ability to process/inhibit distracting information as a function of increasing age. Based on work that mostly focused on unisensory (and, to a great extent, visual) distractor processing, the inhibitory deficit hypothesis (e.g., Hasher & Zacks, 1988; Hasher, Zacks, & May, 1999) was formulated, describing a reduction in inhibitory control with advancing age. Yet, in their review of age-related modulations of the processing of distracting information, Guerreiro, Murphy, and Van Gerven (2010) discuss the findings of more than 150 studies. The authors review age-related changes in distractor processing tasks such as the flanker task or the negative priming paradigm (for a review of the, see Frings, Schneider, & Fox, 2015), in unimodal and crossmodal audiovisual settings. They identify the sensory modality as a critical determinant of the influence of age on selective attention. In particular, in unimodal visual settings, and, to a lesser degree, unimodal auditory settings, age-related decreases in selective attention have been observed. In contrast, in crossmodal settings, this decrease is diminished, and with auditory distracting information, selective attention is mostly preserved (see also Higgen et al., 2020; Poliakoff, Ashworth, Lowe, & Spence, 2006). This stands in line with the call to investigate sensory processing and the ability to inhibit or suppress irrelevant distracting information not only in narrow unisensory settings but also in genuinely crossmodal (and multisensory) settings (Driver & Spence, 1998).

**Fig. 2:**
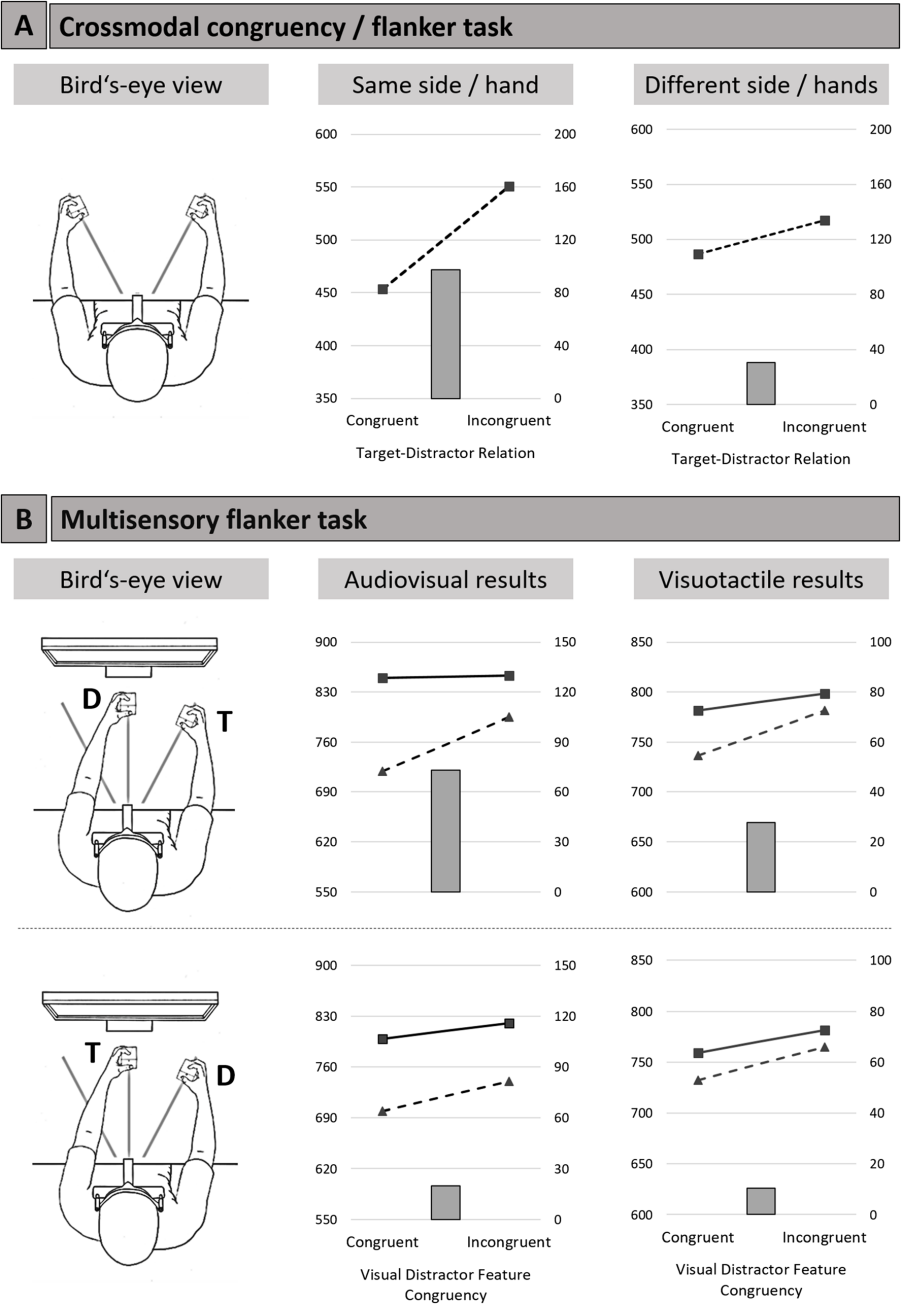
The experimental setup and typical results for the crossmodal congruency/flanker task (**a**) and the multisensory flanker task (**b**). **a** Bird’s-eye view of the arm posture and the respective results from the crossmodal congruency task taken from Spence et al. (2004a, Experiment 1). Line diagram depicts mean RT (left *y*-axis), while the bar diagram depicts the congruency effect (right *y*-axis). **b** Bird’s-eye view of the uncrossed arm posture and the respective results of the audiovisual (Jensen et al., 2019b) and visuotactile (Merz et al., 2019) versions of the multisensory flanker task. Line diagram depicts mean RT (left *y*-axis) for the congruent (triangle) or incongruent (square) auditory/tactile distractor feature, while the bar diagram depicts the interaction score (right *y*-axis). All results in milliseconds. T = target cube; D = distractor cube

In this regard, the crossmodal congruency task (e.g., Spence, Pavani, & Driver, 1998, 2004a, 2004b; for an extensive review, see Spence, Pavani, Maravita, & Holmes, 2008; see also Maravita, Spence, & Driver, 2003) can be seen as the direct extension of the flanker task into a genuinely visuotactile crossmodal setting (see Fig. 2a for an illustration). The participants are tasked with holding a foam cube in each hand, touching two vibrotactile stimulators that are located on the top as well as the bottom of the cube with their index finger (upper location) and thumb (lower location). Two LEDs are incorporated in close spatial proximity to the vibrotactile stimulators. The participants are instructed to make speeded elevation discrimination responses concerning the tactile target stimulus—that is, participants have to indicate if the vibrotactile stimulation was presented on the upper (index finger) or lower location (thumb), irrespective of which hand was stimulated. In each trial, a visual distractor is also presented at one of the four possible locations. Note that, conventionally, the onset of the distractor in this task usually leads the onset of the target by around 30 ms (e.g., Shore, Barnes, & Spence, 2006; Spence et al., 2004a, 2004b).

The congruency effects were calculated by subtracting the performance documented in the congruent trials from that seen in the incongruent trials. In the congruent trials, the visual distractor and tactile target are presented from the same relative (both at the upper or lower) location, whereas in the incongruent trials, the two stimuli indicate different relative locations. Typically, crossmodal congruency effects are most pronounced when the target and distractor stimuli are presented from the same cube/same azimuthal location as compared with a condition in which target and distractor are presented from the other cube (Spence et al., 2004a; for an illustration, see Fig. 2). This holds true even when the participant’s hands are crossed. This is an interesting finding, as it indicates that spatial colocation is more relevant than initial projection of the stimuli in terms of cerebral hemispheres (i.e., with crossed hands, the tactile target and visual distractor are projected into opposite cerebral hemispheres). With hands uncrossed, the crossmodal congruency effects have been shown to decrease with increasing spatial separation between the visual distractor and tactile target stimulus (see Spence et al., 2004b; Spence et al., 2008, for reviews).

A different possible moderator, higher order cognition, does not seem to influence the crossmodal congruency effect. That is, presenting a barrier in between the target and distractor stimulus has no effect on distractor processing (Kitagawa & Spence, 2005, see also Shore & Simic, 2005). In contrast, in a purely tactile task, significant processing changes have been demonstrated due to the presence of a barrier in between the target and distractor hand (Wesslein et al., 2014b). Yet a closer inspection of both studies reveals that such a comparison is unjustified. That is, in the purely tactile task, general distractor interference was observed with and without the barrier (as it is in the crossmodal task), but the level of processing changed due to the introduction of a barrier. Yet in the crossmodal study (Kitagawa & Spence, 2005), a 2 × 2 SR mapping was implemented, thus preventing any analysis of the level of distractor processing, and therefore making any meaningful comparison between the two studies difficult.

Since its first introduction in the closing years of the last century (Spence et al., 1998), the use of the crossmodal congruency task has been increasingly common those researchers wanting to investigate crossmodal information processing. The crossmodal congruency effect results from several factors, including a shift of exogenous spatial attention, a response selection conflict, and/or spatial ventriloquism (Shore et al., 2006). In their extensive review of the crossmodal congruency task, Spence and his colleagues (2008) summarize that it is most likely that each of these factors influence but do not exclusively determine the crossmodal congruency effect (see also Marini, Romano, & Maravita, 2017). The “crossmodal congruency effect is relatively insensitive to various different top-down manipulations” (Spence et al., 2008, p. 34; see also Shore & Simic, 2005). The crossmodal congruency task has subsequently been used to investigate peripersonal space (and its changes due to the usage of, for example, tools, which might be expected to lead to extensions in peripersonal space, e.g., Sengül et al., 2012; Spence, 2011).

The crossmodal congruency task has mostly been operationalized with tactile target and visual distractor stimuli. On occasion, however, the reverse modality pairing has also been investigated, and congruency effects were observed, although diminished in magnitude, as compared with the effects in the original modality pairing (e.g., Spence & Walton, 2005; Walton & Spence, 2004). These asymmetrical results were discussed in terms of possible differences in stimulus salience (although it should be noted that intensity matching across sensory modalities is very difficult if not impossible; see Spence, Shore, & Klein, 2001) or as the result of a bias to automatically allocate attentional resources toward the visual modality during spatial tasks (e.g., Meijer, Veselič, Calafiore, & Noppeney, 2019; Posner, Nissen, & Klein, 1976). Congruency effects in the audiotactile modality pairing have also been observed (Merat, Spence, Lloyd, Withington, & McGlone, 1999; Occelli, Spence, & Zampini, 2009), and crossmodal congruency effects were strongest when the distractor and target stimuli were presented on the same side, comparable with the visuotactile modality pairing (Spence et al., 2004a, b). Interestingly, increasing spatial separation between target and distractor does not exert a significant effect on distractor processing. This stands in contrast to the visuotactile pairings (for an extended discussion, see Kitagawa & Spence, 2006). Yet it is important to note that comparing results across studies are problematic, as differences in stimulus setup, timing, or other factors might impair any reasonable interpretation.

The crossmodal congruency task typically used relative elevation judgments (upper vs. lower discrimination). Yet a nonspatial visuotactile version of the flanker task was also developed, in which not the elevation but the type of stimulus was manipulated and judged (continuous vs. pulsed stimulus presentation; e.g., Holmes, Sanabria, Calvert, & Spence, 2006). In 2010, Frings and Spence (2010) investigated all possible intramodal and crossmodal pairings of the visual, auditory, and tactile modality combinations within two nonspatial congruency experiments. That is, using different rhythms, which had to be classified via keyboard presses, allowed for the presentation of comparable stimuli within all three modalities. Interestingly, the magnitude of the crossmodal congruency effect was mostly influenced by the modality of the target stimulus, and the modality of the distractor stimulus did not have a significant influence on the crossmodal congruency effects. More precisely, congruency effects for auditory targets were smallest compared with visual and tactile targets, as the congruency effects between visual and tactile targets did not differ consistently between the two experiments. Overall, this puts emphasis on the response-relevant target modality and shows once again the specificity and importance of crossmodal investigations. Yet future research should aim to differentiate between the different levels of distractor processing and, for example identify if modulations of higher order cognitions in truly crossmodal tasks result in similar changes as shown for unisensory processing (see Wesslein et al., 2014b).

### Multisensory distractor processing in the flanker task

The literature reviewed so far has not included any genuinely multisensory selection situations—by which we mean that no has the situation in which the target and/or the distractor are multisensory—that is, the stimuli are specific composites of feature information from different modalities. For example, while talking to a colleague at a conference party, we look at her to see as well as listen to her talking, while ignoring another colleague standing right beside her, and who is herself in a lively conversation.

To investigate distractor processing in a multisensory setting, we designed a multisensory variant of the flanker paradigm (e.g., Jensen, Merz, Spence, & Frings, 2019b; Merz, Jensen, Spence, & Frings, 2019; for an illustration, see Fig. 2b). The target as well as the distractor were composites of either the audiovisual (Jensen et al., 2019b) or visuotactile (Merz et al., 2019) feature combination, and the target and distractor stimulus were each presented from a multisensory cube. Importantly, the two features of each stimulus were presented simultaneously from one stimulus location (the multisensory cube), therefore ensuring that they are temporally and spatially aligned, as these represent a necessary precondition for multisensory integration/processing to occur (Spence, 2013; Stein & Meredith, 1993; Stein & Stanford, 2008). The response relevant target object was constant within one experimental block, allowing for the allocation of (covert) attention towards the target stimulus. The experiments were designed with a 2 × 2 SR mapping, and the target stimulus that had to be identified (with either a left or right foot-pedal press) were two specific combinations of a visual (color) feature and an auditory (frequency) or tactile (intensity) feature (e.g., “red–high intensity” and “blue–low intensity” combinations; Merz et al., 2019). To ensure that participants responded to the multisensory feature combination, not just to one of the unimodal features, the reversed feature combinations were also presented as catch trials in one-fifth of the trials (e.g., “red–low intensity” and “blue–high intensity” combinations). Those four feature combinations were also used for the distractor stimulus, and furthermore, a response-neutral feature was added in each modality (green color for vision, middle intensity for touch), to underline the irrelevance of the distractor stimulus.

This task design allows for the investigation of the way in which the features of the distractor were processed. Hereby, two general ways in which the distractor feature information is processed might occur: The distractor features are processed in isolation—in other words, unisensory/independent processing of the distractor features occurs. Alternatively, multisensory distractor processing occurs—that is, the features of the distractor are not presented in isolation and are combined somewhere during information processing. The two different processing strategies can be differentiated at the stage of data analysis. If unisensory distractor processing occurs, significant main effects of congruency would be expected for each modality, but crucially, there should be no interaction between congruency and modality. That is, the typical congruency effects are elicited by the distractor, yet the congruency of one feature did not change with changes in the congruency in the other feature. In contrast, if multisensory distractor processing occurs, a significant interaction would be evidenced, as this indicates that the processing of one feature is not independent from the identity of the other feature.

In our research (e.g., see Jensen et al., 2019b; Merz et al., 2019), we systematically manipulated the spatial attentional resources directed toward the distractor or target stimulus by presenting either the distractor or target stimulus in the center of participant's gaze (see Fig. 2b). Interestingly, only when spatial resources were directed toward the distractor (i.e., the distractor was presented in the participant’s gaze), multisensory distractor processing occurred. In fact, when directing spatial resources toward the target and decrease spatial attention resources toward the distractor by increasing the eccentricity of the distractor from the participant’s gaze, the multisensory distractor processing turned gradually into unisensory distractor processing (in other words, the interaction weakened and disappeared). This was true for the audiovisual (Jensen et al., 2019b) as well as visuotactile (Merz et al., 2019) modality combinations, thus indicating a general, modality-independent influence of attention on multisensory distractor processing (for a critical discussion about the concept of attention, see Hommel et al., 2019).

In subsequent studies, we explored the relevance of the attentional set in multisensory selection situations (Jensen, Merz, Spence, & Frings, 2019a). We further observed that higher order cognition did not affect multisensory distractor processing, seemingly contrasting with the evidence that has been obtained from the tactile modality (Merz, Jensen, Burau, Spence, & Frings, 2020). This underlines the importance of investigating truly multisensory selection situations as evidence from strictly unisensory or crossmodal task setting might not easily be transferred to a multisensory situation. In another study (Jensen, Merz, Spence, & Frings, 2019c), we used the multisensory flanker task to investigate the processing level of the multisensory target (not distractor). In this study, participants conducted the multisensory flanker task in a first step so to ensure that multisensory processing of the target stimulus occurred. The processing level of the target stimulus was subsequently investigated with the help of the aftereffects of target processing by manipulating congruency along the perceptual and/or response level in a subsequent crossmodal task. The results indicated that multisensory target processing occurred mostly at the perceptual level.

To summarize, since its first introduction in 1974, the flanker task was, and still is, a helpful tool with which to investigate the processing of irrelevant information not just in unisensory, but also in crossmodal and, more recently, in multisensory settings. The comparison of the results between the different settings indicate a clear message: A simple generalization of result from unisensory settings to crossmodal and/or multisensory settings should not be assumed. This summary indicates that each task setting is unique on its own, and it opens the question of whether each task setting actually tackles independent theoretical questions or if the common underlying mechanisms have not been detected yet.

## A multisensory perspective on selection: Open questions and future directions

After its first introduction almost 50 years ago (B. A. Eriksen & C. W. Eriksen, 1974), the flanker task has been successfully adapted to investigate the cognitive mechanisms underlying selection in crossmodal and, more recently, multisensory situations. With the newly developed multisensory version of the flanker task (Jensen et al., 2019b; Merz et al., 2019), researcher have a unique ability to investigate the nature of multisensory processing. Please note that the term “multisensory processing” is used deliberately, rather than the more common and specific term of “multisensory integration" (e.g., Stein & Meredith, 1990; Stein & Stanford, 2008), as multisensory integration is possibly too narrowly defined to describe the processes observed in (adaptations of) the multisensory flanker task.^1^ The multisensory flanker task turns its focus from the task-relevant information, which is important for current (behavioral) goals, to the task-irrelevant information, which has to be ignored to successfully achieve these (behavioral) goals. This change in focus opens up a number of tantalizing new possibilities to further the understanding of multisensory processing in general. In fact, the multisensory flanker task enables us for the first time to investigate the processing and internal representation of *multisensory* information that is not selected for action and interferes with current task goals.

### Multisensory selection and the flanker task

The fact that the multisensory flanker task investigates task-irrelevant distracting information introduces a new possibility to investigate one of the most controversial topics in the multisensory processing literature—that is, the importance of attention for multisensory processing. The data pattern concerning the interplay between attention and multisensory processing is inconsistent, as some results indicate that multisensory processing is modulated by attention (e.g., Alsius, Navarra, Campbell, & Soto-Faraco, 2005; Alsius, Navarra, & Soto-Faraco, 2007), whereas other studies observe multisensory processing to be automatic (e.g., Bertelson, Vroomen, de Gelder, & Driver, 2000; Santangelo & Spence, 2007). This conflicting evidence has been unified by frameworks emphasizing selection difficulty, learned association, spatial configurations, or cognitive load (and salience), as key factors determining the influence of attention for multisensory processing (e.g., De Meo, Murray, Clarke, & Matusz, 2015; Fiebelkorn, Foxe, & Molholm, 2010; Navarra, Alsius, Soto-Faraco, & Spence, 2010; Santangelo & Macaluso, 2012; Talsma, 2015; Talsma, Senkowski, Soto-Faraco, & Woldorff, 2010; Tang, Wu, & Shen, 2016). Yet these studies and theoretical ideas were mostly based on studies investigating multisensory target processing (i.e., multisensory information that is attended and/or responded to), and therefore some attention was directed toward these stimuli by default. In contrast, in the multisensory variant of the flanker task, the distractor stimulus is irrelevant and therefore, no attention is voluntarily directed toward the distractor stimulus by default.

In fact, our first results with the multisensory flanker task were in line with the idea that (spatial) attention is necessary for multisensory processing to occur, as the distractor features were only combined if the distractor was presented at the center of participant’s gaze (Jensen et al., 2019b; Merz et al., 2019; see also Fig. 2). In a way, this fits Treisman’s formulation of attention being the glue that binds single features into (multisensory) object representations (Treisman & Gelade, 1980; for a discussion of feature integration theory in the multisensory world, see Spence & Frings, 2020). Yet attention was only manipulated along the spatial dimension—that is, the distractor (or target) was either presented inside or outside of the participant’s gaze. attention is not limited to the spatial domain, and future research should identify whether the present results can be generalized to manipulations of nonspatial attention (Duncan, 1984; Found & Müller, 1996; Müller, Heller, & Ziegler, 1995).

Furthermore, the results might also fit with those accounts describing a moderating influence of factors such as selection difficulty or cognitive load and salience on the interplay between attention and multisensory processing (e.g., De Meo et al., 2015; Navarra et al., 2010; Talsma, 2015; Talsma et al., 2010; Tang et al., 2016). Yet, to this point, it is an open question as to how the multisensory flanker task qualifies along these proposed dimensions. Therefore, future research needs to systematically manipulate factors like stimulus salience, task difficulty, and cognitive load to see if these theoretical ideas are generalizable to include multisensory processing of task-irrelevant stimuli.

Another crucial question concerns the processing stage at which multisensory processing in selection situations might occur. In fact, adaptations of the flanker task are equipped to provide insight concerning the multisensory processing of not just the distractor but also the target. By combining the flanker task with the logic of crossmodal aftereffects, we have identified that the multisensory target (not distractor) is processed mainly on the perceptual level (Jensen et al., 2019c). If this translates to the processing of multisensory distractors is an open question at this point in time. In fact, by using a more elaborate task set like the 4 × 2 SR mapping, the processing level at which distractor processing occurs can presumably be identified in the future.

### Multisensory selection beyond the flanker task

The previous section outlines some of the most promising and pressing theoretical questions for which the multisensory flanker task likely proves to be insightful. Yet the flanker task has a relatively narrow focus on the “online” effect of distractor processing during selection. That is, the focus is on the immediate effect of the processing of the distractor stimulus. Therefore, the flanker task helps to investigate the processing of the distracting information in the short time while participants respond to the target stimulus. However, the effect of selection is not limited to this narrow window of time, and sequential effects like the Gratton (Gratton, Coles, & Donchin, 1992; for a review, see Verguts & Notebaert, 2008), negative priming (Neill, 1997; Tipper, 2001; for a review, see Frings, Schneider, & Fox, 2015), or distractor response binding effect (Frings, Rothermund, & Wentura, 2007) take a closer look at the direct processing consequences of selection.

Extending the multisensory perspective from the “online” flanker task investigations to short-term sequential selection paradigms, as well as to long-term learning investigations, is a necessary future step to gain a holistic understanding of selection in a multisensory world. What happens directly after the multisensory target stimulus is successfully selected against the distracting information and responded to? Evidence from mostly unisensory, and some crossmodal, investigations suggest that this initiates some kind of a control process, as the “online” congruency effect in flanker tasks are reduced after incongruent compared with congruent trials (the so-called Gratton effect; for an elaborate discussion, see Verguts & Notebaert, 2008). What is more, ignoring distracting information in one trial impairs responding to this previously ignored stimulus in the next trial (for a detailed discussion concerning the underlying mechanisms of this negative priming effect, see Frings et al., 2015). This indicates a profound impact of stimulus selection on subsequent short-term processing. The question arises as to how these mechanisms transfer to truly multisensory situations. Is there a change in the cognitive control process when the distractor was processed as a multisensory stimulus compared with when the stimulus sensory features are processed independently? What exactly is ignored during multisensory selection? Is every feature on its own ignored, or is only the specific multisensory distractor combination impaired during multisensory selection? Eventually, these discussions should not be limited to the short-term consequences investigated with these sequential selection paradigms (typically, the evidence from these paradigms is limited to a few seconds after initial selection; e.g., Frings, Schneider, & Fox, 2015; Verguts & Notebaert, 2008). In fact, if and how possible short-term associations (during selection) can manifest themselves in long-term memory traces is a controversial topic (e.g., Abrahamse, Jiménez, Verwey, & Clegg, 2010; Cleeremans, Destrebecqz, & Boyer, 1998; Logan, 1988; Verwey, Shea, & Wright, 2015), and taking an explicit multisensory perspective on this debate should be a future undertaking.

One final point within this review is directed toward the underlying purpose of selection. In fact, it is commonly argued that the most important reason for selection is to act on the selected (and not the ignored) stimulus, resulting in the idea of “selection for action” (e.g., Allport, 1987). In fact, following recent developments in action control, these sequential selection processes are the result of binding information (about the stimuli, the executed response and the [sensory] effects; Moeller, Pfister, Kunde, & Frings, 2019) into an event file (see Hommel, 2004). This event file is then subsequently retrieved in the following trial (Frings et al., 2020). The distinction between the binding/integration process on the one side, and the subsequent retrieval process on the other (which can be experimentally discriminated; e.g., Laub, Frings, & Moeller, 2018), introduces new questions about the processing of multisensory information. At which stage (the integration/binding or the retrieval process)? Is attention, which seems to have an impact on multisensory target as well as distractor processing (e.g., Jensen et al., 2019b; Merz et al., 2019), influencing the integration, the retrieval, or both? In which exact way are the multisensory features associated with the to-be-executed response? Is this different under conditions of independent, unisensory processing as compared with multisensory processing? These questions are just the tip of the iceberg as far as the investigations in this area may proceed in the future. In fact, we believe that the multisensory flanker task can be seen as the baseline task from which these (and many more) theoretical questions can be tackled in the future.

## Conclusion

Since its first introduction in 1974, the flanker task (B. A. Eriksen & C. W. Eriksen, 1974) has been used to investigate the cognitive underpinnings of selection, not just in unisensory but also in crossmodal and, more recently, in multisensory situations. By reviewing the existing literature, it soon becomes clear that the simple generalization of evidence from one sensory modality to the other (e.g., Chan et al., 2005; Driver & Grossenbacher, 1996; Fox, 1998; Miller, 1991), or from unisensory to crossmodal and/or multisensory settings, falls short when explicitly tested (e.g., Guerreiro et al., 2010). Furthermore, it is argued that adaptations of the crossmodal, and especially the multisensory version of the flanker paradigm, have several important qualities (e.g., investigation of the processing level of irrelevant information). Therefore, this task should be used to investigate not just multisensory processing on its own, but how multisensory processing is affecting selection in the multisensory setting. The interplay of attention and multisensory processing is perhaps best studied with experimental tasks that can disentangle task relevance from attention. Hence, multisensory variants of the flanker tasks will further our understanding of multisensory selection in general. In addition, literally nothing is known about sequential effects of multisensory distractor processing and again the flanker task (or variants thereof) is very well suited to investigate what happens to the representation of a multisensory distractor stimulus (and whether it can affect subsequent behavior). In other words, selection research has to embrace the reality that we act and select in a multisensory world, and the flanker task will be one of the tools best suited to pursue multisensory selection research in the future.

## Footnotes

1 In our view, multisensory processing describes any multisensory phenomenon that includes multisensory integration, but is not limited to it. In fact, we see the term multisensory integration as being too narrow, as the wording might imply (depending on your previous experience with that topic) that it only describes neural processes or processing on early perceptual levels. Yet it is too early to be able to make these specific differentiations, and therefore we use the more general “multisensory processing” term, which is also suggested by Stein et al. (2010), who explicitly discuss the problems of the “multisensory integration” term.
